# HIV-1 Tat Activates Akt/mTORC1 Pathway and *AICDA* Expression by Downregulating Its Transcriptional Inhibitors in B Cells

**DOI:** 10.3390/ijms22041588

**Published:** 2021-02-04

**Authors:** Burkitkan Akbay, Diego Germini, Amangeldy K. Bissenbaev, Yana R. Musinova, Evgeny V. Sheval, Yegor Vassetzky, Svetlana Dokudovskaya

**Affiliations:** 1 CNRS UMR9018, Institut Gustave Roussy, Université Paris-Saclay, 94805 Villejuif, France; akbayburkitkan@gmail.com (B.A.); germinidiego@gmail.com (D.G.); Yegor.VASSETZKY@gustaveroussy.fr (Y.V.); 2 Department of Molecular Biology and Genetics, Faculty of Biology and Biotechnology, Al-Farabi Kazakh National University, Almaty 050040, Kazakhstan; Amangeldy.Bisenbaev@kaznu.kz; 3 Scientific Research Institute of Biology and Biotechnology Problems, Al-Farabi Kazakh National University, Almaty 050040, Kazakhstan; 4 Koltzov Institute of Developmental Biology of Russian Academy of Sciences, 119991 Moscow, Russia; musinova.yana@gmail.com; 5 Belozersky Institute of Physicochemical Biology, Moscow State University, 119899 Moscow, Russia; esheval@gmail.com

**Keywords:** HIV-1, Tat, Akt/mTORC1 pathway, AICDA, B cells

## Abstract

HIV-1 infects T cells, but the most frequent AIDS-related lymphomas are of B-cell origin. Molecular mechanisms of HIV-1-induced oncogenic transformation of B cells remain largely unknown. HIV-1 Tat protein may participate in this process by penetrating and regulating gene expression in B cells. Both immune and cancer cells can reprogram communications between extracellular signals and intracellular signaling pathways via the Akt/mTORC1 pathway, which plays a key role in the cellular response to various stimuli including viral infection. Here, we investigated the role of HIV-1 Tat on the modulation of the Akt/mTORC1 pathway in B cells. We found that HIV-1 Tat activated the Akt/mTORC1 signaling pathway; this leads to aberrant activation of activation-induced cytidine deaminase (*AICDA*) due to inhibition of the AICDA transcriptional repressors *c-Myb* and *E2F8*. These perturbations may ultimately lead to an increased genomic instability and proliferation that might cause B cell malignancies.

## 1. Introduction

HIV-1 trans-activator of transcription (Tat) is one of the key viral regulatory proteins essential for HIV-1 replication, establishment of infection and virus reactivation [1,2]. Tat is a small protein of 86–102 amino acids (depending on the viral strain) with a predicted molecular weight of 14–16 kDa. HIV-1 predominantly infects T cells and macrophages, and Tat is expressed in productively infected cells. Tat can be released from infected cells into blood, where it can penetrate many cell types due to its protein transduction domain [3,4,5,6]. Therefore, Tat may affect both infected and non-infected cells including B cells, which may participate in the oncogenesis of HIV-associated malignancies [7,8].

HIV infection is associated with an elevated risk of developing AIDS-defining cancers, including a number of B-cell lymphomas [9,10]. Even though a current antiretroviral therapy is quite efficient, a high incidence of HIV-1-associated malignancies persists and the mechanism of development of these diseases is largely unclear [11]. Tat, which persists in the blood of HIV-1 patients even under the antiretroviral therapy [12], can penetrate uninfected cells and modulate host gene expression, having direct or indirect oncogenic effects on B cells [13,14]. We have recently demonstrated that Tat induces oxidative stress and DNA damage in peripheral blood lymphocytes [15], and induces repositioning of the *MYC* locus on chromosome 8 in the close proximity to the *IGH* locus on the chromosome 14, thereby increasing the chances of the oncogenic translocation t (8;14) characteristic of Burkitt lymphomas [12]. Deregulation of the Akt/mTORC1 pathway could be the major contributor to the development of AIDS-related lymphomas (ARLs), as various studies have shown the hyper-activation of this signaling pathway in different subgroups of ARLs [13,16,17,18,19,20,21]. 

The mTORC1 pathway integrates signals from many intracellular and extracellular cues: growth factors, amino acids, energy, oxygen, DNA damage and infectious agents, including viruses [22,23]. The Akt/mTORC1 axis is activated in response to growth factors, cytokines, insulin and DNA damage [23,24]. mTORC1 phosphorylates many targets, among them eukaryotic initiation factor 4E-binding protein 1 (4E-BP1) and p70S6 kinase 1 (S6K1), promoting the synthesis of three major cell constituents: proteins, nucleotides and lipids [25,26,27,28,29].

mTORC1 plays a central role in the metabolism, differentiation and effector functions of immune cells [30,31] and is essential for the development of B cells as well as for the antibody production [32,33,34]. mTORC1 inactivation leads to decreased expression of the activation-induced cytidine deaminase (*AICDA*), which impairs the generation of high-affinity antibodies [34,35]. The physiological function of *AICDA* is to promote the somatic hypermutation and class-switch recombination (CSR) of the immunoglobulin (Ig) loci. Aberrant expression of *AICDA* and its involvement in lymphomagenesis have been observed in different subtypes of B-cell lymphomas [36,37]. Our recent results demonstrate that HIV-1 Tat can induce aberrant expression of *AICDA* in peripheral blood B cells from HIV-infected individuals [38].

We hypothesized that Tat could modulate the Akt/mTORC1 pathway in B cells, and here, we demonstrated that in B cells, HIV-1 Tat not only induced reactive oxygen species (ROS) production and DNA damage, but also activates the Akt/mTORC1 pathway, resulting in *AICDA* overexpression via mTORC1-dependent inhibition of *AICDA* repressors *c-Myb* and *E2F8*.

## 2. Results

### 2.1. HIV-1 Tat Activates the AKT/mTORC1 Pathway in B Cells as a Response to ROS-Induced DNA Damage

**Figure 1:**
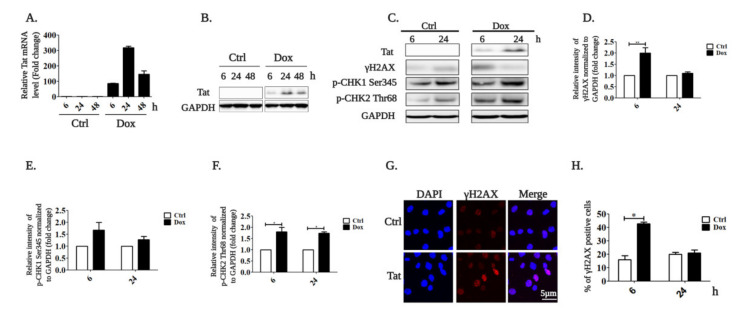
HIV-1 Tat induces DNA damage in B cells. (**A**) Doxycycline-inducible Tat-expressing RPMI-8866 cells were treated (Dox) or not (Ctrl) with 1 µg/mL doxycycline for 6, 24 and 48 h. Tat expression was analyzed using qRT-PCR and quantified by the 2^−ΔΔct^ method comparing the expression of the target gene in cells treated with Dox with the untreated control (set as 1) after normalization with *GAPDH* gene expression. (**B**) Doxycycline-inducible Tat-expressing RPMI-8866 cells were treated as in (**A**) for 6 and 24 h. Tat expression was identified with an appropriate antibody. (**C**) RPMI-8866 cells were treated as in (**A**) and analyzed 6 and 24 h after induction. Tat, γ-H2AX and p-CHK2 Thr68 were detected by Western blotting with appropriate antibodies. (**D**) Intensities of γ-H2AX, (**E**) p-CHK1 Ser345 and (**F**) p-CHK2 Thr68 bands were quantified with ImageJ software in Dox-treated cells compared to the untreated control (set as 1) after normalization with GAPDH band intensities, which was used as the loading control. (**G**) γH2AX was immuno-stained in doxycycline-inducible Tat-expressing RPMI-8866 cells treated as in (**A**) for 6 h. Nuclei stained with DAPI are in blue, and γ-H2AX staining is shown in red. (**H**) Images from D were analyzed using the ImageJ software to evaluate the percentage of cells with the γH2AX staining. All data represent the results of three independent experiments and are expressed as the mean ± SEM. The statistical significance was calculated vs. untreated controls; * 0.01 < *p* < 0.5, ** 0.001 < *p* < 0.01.

To study the function of HIV-1 Tat in B cells, we recently generated an inducible model system using the immortalized lymphoblastoid B cell line (RPMI-8866), where Tat expression can be induced by doxycycline [39]. Tat expression initiates after 6 h of treatment with 1 µg/mL doxycycline and peaks at 24 h both at the mRNA (Figure 1A) and protein level (Figure 1B).

We and others have recently shown that HIV-1 Tat induces oxidative stress and DNA damage in different cell types [15,40,41]. In order to verify if HIV-1 Tat activates the DNA damage in our inducible model, we checked the levels of different DNA damage markers. We found that DNA damage was induced 6 h after HIV-1 Tat expression induction, as demonstrated by the increased level of γH2AX (Figure 1C,D) and phosphorylation of CHK1 (p-CHK1 Ser345) and CHK2 (p-CHK2 Thr68) (Figure 1C,E,F). We confirmed this by immunofluorescence staining of γH2AX (Figure 1G). We found that 42.7 ± 1.2% of cells with induced Tat expression exhibited γH2AX staining compared to 15.8 ± 3% of non-induced control cells (Figure 1H). The DNA damage is then rapidly repaired by the DNA damage response system (DDR), as previously demonstrated [12,15].

**Figure 2:**
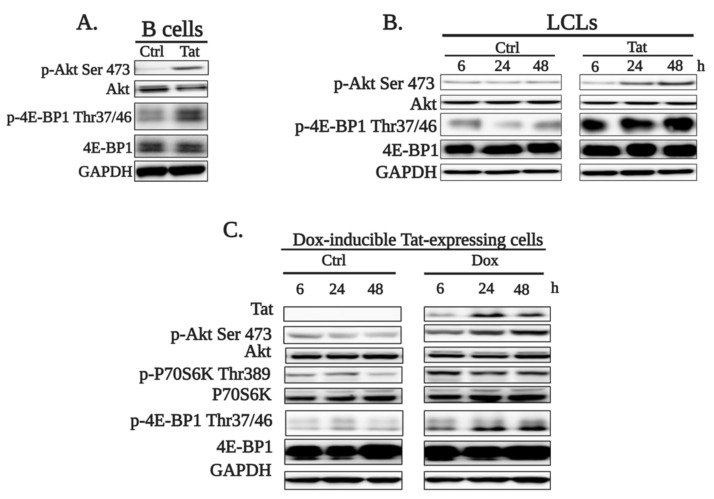
HIV-1 Tat activates the Akt/mTORC1 pathway in B cells. (**A**) Primary B cells purified from the blood of healthy donors were left untreated (Ctrl) or treated (Tat) with 250 ng/mL Tat for 48 h. (**B**) Immortalized lymphoblastoid cell lines (LCLs) were treated with 250 ng/mL Tat for 6, 24 and 48 h. (**C**) Tat expression was induced (Dox) in doxycycline-inducible Tat-expressing RPMI-8866 cells by treating with 1 µg/mL doxycycline for 6, 24 and 48 h. Tat, Akt and its phosphorylated form (p-Akt Ser473), P70S6K and its phosphorylated form (p-P70S6K Thr389) and 4E-BP1 as well as its phosphorylated form (p-4E-BP1 Thr 37/46) were detected by Western blotting with specific antibodies. GAPDH was used as the loading control. Representative images of three independent experiments are shown. The image quantification and statistical analysis are shown in Figure S1.

Activation of the Akt/mTORC1 pathway is one of the consequences of the DDR. In order to determine if Tat treatment affects the Akt/mTORC1 pathway, we used three cell models: primary B cells from healthy donors, immortalized lymphoblastoid cell lines (LCLs, RPMI-8866) and the doxycycline-inducible Tat-expressing RPMI-8866 cell line. Primary B cells and LCLs were treated with 250 ng/mL Tat for 6, 24 and 48 h (B cells) by mimicking the penetration of secreted Tat protein from infected cells, and Akt phosphorylation at serine 473 and the phosphorylation 4E-BP1 (p-4E-BP1 Thr 37/46), a marker of mTORC1 activity, were analyzed. We found that exogenous Tat treatment significantly increased the phosphorylation of Akt Ser473 and 4E-BP1 Thr37/46 starting from 24 h, indicating that Tat activates the Akt/mTORC1 signaling pathway (Figure 2A,B and Figure S1A–D). Activation of Akt and mTORC1 was also confirmed in doxycycline-inducible Tat-expressing RPMI-8866 cells, where phosphorylation of Akt Ser473, 4E-BP1 Thr37/46 and P70S6K Thr389 was increased in response to Tat induction starting 6 h after Dox treatment (Figure 2C and Figure S1E–G). Altogether, these results demonstrate that Tat activates the Akt/mTORC1 pathway in B cells.

**Figure 3:**
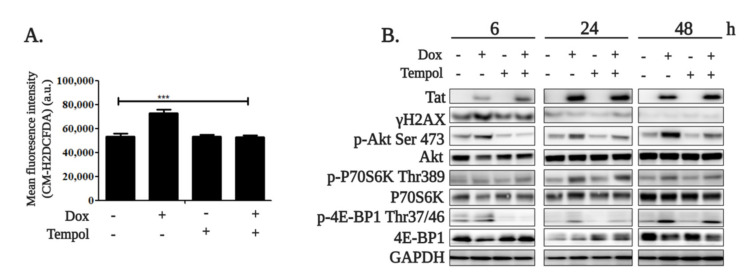
Activation of the Akt/mTORC1 axis is dependent on the DNA damage induced by reactive oxygen species (ROS) production. (**A**) Tat expression was induced in the RPMI-8866 cells by treating with 1 µg/mL doxycycline for 6 h (Dox) in the presence or absence of 80 µM Tempol. ROS production was measured by flow cytometry after CM-H2DCFDA staining as described in the Materials and Methods. Staining intensity is represented as the mean fluorescence in arbitrary units. (**B**) Tat and phosphorylated and non-phosphorylated forms of Akt, P70S6K and 4E-BP1 were analyzed in Tat-expressing cells, in cells treated with Tempol at a final concentration. The results of three independent experiments are expressed as the mean ± SEM. The statistical significance was calculated vs. untreated controls; *** *p* < 0.001

It is known that HIV-1 Tat induces reactive oxygen species (ROS) production and DNA damage within several hours after penetration in various cell types, including B cells [15,40,41]. ROS production can activate the Akt/mTORC1 pathway. Therefore, we checked if it is the case in our model. Tat expression was induced in the presence or absence of 80 μM Tempol, a ROS scavenger, and ROS level was measured 6 h after induction. ROS levels were three times higher in HIV-1 Tat-expressing cells compared to the untreated cells. Treatment with Tempol completely abolished HIV-1 Tat-induced ROS production (Figure 3A). Consistently, increased ROS levels were associated with increased γH2AX staining 6 h after *Tat* induction. Consistently, Tempol treatment decreased γH2AX levels (Figure 3B). Akt/mTORC1 pathway activation was also prevented by Tempol (Figure 3B). Interestingly, DNA damage was decreased at 24 and 48 h in all conditions regardless of the treatments, as indicated by a significant reduction of γH2AX level. The Akt/mTORC1 pathway, however, remained activated up to 48 h after Tat induction. These results suggest that once activated in response to DNA damage caused by ROS, Akt/mTORC1 pathway activity is maintained by Tat, even if the DNA damage signal decreases.

### 2.2. Tat Induces AICDA Expression by Downregulating c-Myb and E2F8 in an mTORC1-Dependent Manner

**Figure 4:**
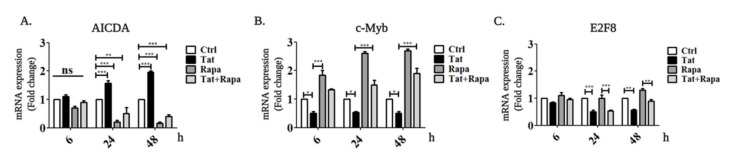
Tat induces activation-induced cytidine deaminase (*AICDA*) overexpression by inhibiting its repressors in an mTORC1-dependent way. Tat expression was induced in the RPMI-8866 cells by addition of 1 µg/mL doxycycline for 6, 24 and 48 h, followed by cells being treated with 200 nM of rapamycin (Tat + Rapa) or left untreated (Tat). RPMI-8866 cells not expressing Tat were also treated with 200 nM rapamycin (Rapa) for the indicated times or left untreated (Ctrl). *AICDA* (**A**), *c-Myb* (**B**) and *E2F8* (**C**) mRNA expression levels were analyzed at different time points by qRT-PCR and quantified using the 2^−ΔΔct^ method comparing the expression of the target gene in Tat-induced, rapamycin-treated and Tat-induced + rapamycin-treated cells with the untreated control (set as 1) after normalization with *GAPDH* gene expression. Results are obtained from three independent experiments. The statistical analyses were carried out by the one-way ANOVA test. All data are expressed as the mean ± SEM. The statistical significance was calculated between groups; *** *p* < 0.001, ** 0.001 < *p* < 0.01, * 0.01 < *p* < 0.5. ns comes for not segnificant.

We have previously demonstrated that *AICDA* transcription was induced in B cells from healthy donors incubated with HIV-1 Tat and in B cells from HIV-patients, yet how this activation occurs was not clear [38]. mTORC1 positively regulates *AICDA* expression [42]; therefore, we hypothesized that HIV-1 Tat-induced *AICDA* transcription in B cells is dependent on the mTORC1 pathway. To test this hypothesis, we analyzed *AICDA* expression after induction of *Tat* expression in the absence or presence of the mTORC1 inhibitor rapamycin. *AICDA* expression was upregulated 1.7-fold and 2-fold 24 and 48 h after Tat induction, respectively (Figure 4A). mTORC1 inhibition by rapamycin robustly downregulated *AICDA* expression even in the presence of Tat, demonstrating the dependency of Tat-derived AICDA upregulation from the mTORC1 pathway (Figure 4A).

**Figure 5:**
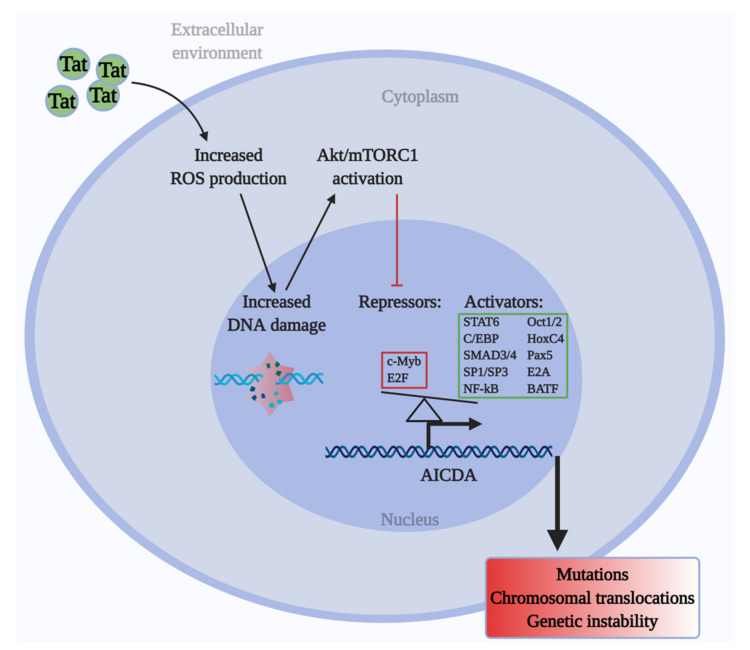
Oncogenic effects of HIV-Tat in B cells. Tat increases the ROS production [15], leading to the induction of DNA damage and activation of the Akt/mTORC1 pathway. This results in the increased expression of *AICDA* due to the mTORC1-dependent inhibition of its transcriptional repressors. Deregulated expression of *AICDA* increases the possibility of increased mutations and chromosomal translocations causing genetic instability in B cells, leading to the emergence of B-cell malignancies.

To elucidate how mTORC1 regulates *AICDA* in Tat-expressing B cells, we analyzed the expression of known transcriptional regulators of *AICDA* (10 activators and 2 repressors) in the presence or absence of Tat and rapamycin. The expression of all transcriptional activators was essentially non-affected in all conditions tested (Figure S2), while both repressors, *c-Myb* and *E2F8*, were found to be strongly inhibited by HIV-1 Tat with or without rapamycin (Figure 4B–C). In the absence of Tat, treatment with rapamycin strongly induced *c-Myb* expression, but did not significantly affect *E2F8*. These results demonstrate that Tat upregulates *AICDA* expression by downregulating the expression of its repressors *c-Myb* and *E2F8* in an mTORC1-dependent manner (Figure 5).

## 3. Discussion

The Akt/mTORC1 pathway is important for the successful entry, integration and replication of HIV-1 in peripheral blood lymphocytes, memory T cells and dendritic cells [43,44,45,46,47,48]. Akt/mTORC1 activity can also be modulated by individual HIV-1 proteins. For example, mTORC1 can be activated by treatment with HIV-1 Nef and Env in HEK293T, endothelial and dendritic cells [43,49]. Activation of Akt/mTORC1 by Tat has been documented in HeLa, Jurkat and peripheral blood mononuclear cells [45,48]. To the best of our knowledge, however, no studies have been conducted to examine the regulation of mTORC1 by HIV-Tat in B cells, even though they are the main part of the immune system.

Here, we demonstrated that, in agreement with previous studies conducted on other cell models [15,40,41], Tat induced ROS production and DNA damage in B cells, which resulted in hyper-activation of the Akt/mTORC1 pathway. The Akt/mTORC1 pathway is often upregulated in AIDS-related B-cell lymphomas [13,16,17,18,19,20,21]. Tat is constantly present in detectable concentrations in patients’ blood serum [12,50] and can potentially enter any cell in a human body due to its cell penetration domain [51]. Tat’s presence leads to transient ROS activation and DNA damage, which in the long term may induce chromosomal aberrations typical of lymphomas [15]. In addition, Tat can probably remotely regulate mTORC1 activity in many non-infected cells.

Our group previously described that Tat could remodel the nuclear architecture, leading to MYC-IGH loci proximity [12] and *AICDA* overexpression in B cells from healthy donors and from HIV-infected patients [38]. Thus, Tat can predispose to the MYC-IGH t(8;14) chromosomal translocation, a hallmark of Burkitt lymphoma. At the same time, the mechanisms leading to AICDA activation are unexplained. *AICDA* expression is significantly elevated in PBMCs of HIV-infected individuals who later developed AIDS-associated lymphomas, especially in those who developed Burkitt’s lymphoma [52]. Elevated *AICDA* expression is also found in lymph node tissue samples from lymphoma patients, and it is suggested to be used as a marker of unfavorable outcomes in diffuse large B-cell lymphoma patients treated with thermotherapy [53]. HIV proteins could also contribute to the development of lymphomas in HIV-infected patients. Indeed, HIV-Nef significantly promotes *AICDA* expression along with *MYC* expression in Burkitt lymphoma [54].

An increasing number of studies have demonstrated a positive relation between mTORC1 activity and *AICDA* expression. Deletion of the mTORC1 component Raptor in B lymphocytes results in decreased *AICDA* expression, which was associated with the loss of BCL6, a key transcriptional regulator involved in germinal center fate [35]. Moreover, inhibition of mTORC1 by rapamycin results in the decreased AICDA protein level, leading to decreased antibody class-switching [55]. mTORC1 is found to be highly activated and *AICDA*, its transcriptional activator *PAX*5, and *BCL6* are overexpressed in a population of atypical memory B cells in patients with active lupus [56]. Therefore, it is possible that mTORC1 regulates *AICDA* expression either through BCL6 or through direct transcriptional regulators of AICDA. Consistently, we have found that mTORC1 positively regulated *AICDA* expression in B cells. Even though we did not observe the overexpression of *PAX*5, we found that mTORC1 promotes *AICDA* expression by inhibiting its two major repressors, c-Myb and E2F8.

Our data suggest that extracellular Tat penetrates B cells and induces ROS production, leading to DNA damage within a short time. Increased DNA damage is further sensed by the Akt/mTORC1 pathway. Activated mTORC1 inhibits the repressors of *AICDA*, *c-Myb* and *E2F8*, leading to the disruption of the balance between the transcriptional activators and repressors of *AICDA*, which ultimately results in the overexpression of *AICDA*. Deregulated *AICDA* expression may increase the possibilities of mutations and chromosomal translocations underlying B-cell malignancies.

## 4. Materials and Methods

### 4.1. Cell Lines and Blood Samples

The lymphoblastoid cell line RPMI-8866 and its Tat-expressing variant [39] were cultured at 37 °C in RPMI 1640-GlutaMAX medium (#61870-010, Gibco, Bourgoin-Jallieu, France) supplemented with 10% fetal bovine serum (#10270-106, Gibco, France) and 1% penicillin/streptomycin (#15140-122, Gibco, France). 

Peripheral blood mononuclear cells were isolated from whole blood by Pancoll (PAN Biotech, Aidenbach, Germany, lymphocyte separation medium). B cells were then purified using the MagniSort B cells enrichment kit II (Thermo Fisher Scientific, Bourgoin-Jallieu, France). Purified B cells were then kept at 37 °C in the culture medium described above.

### 4.2. Treatments

Doxycycline (#3447, Sigma-Aldrich, Saint Louis, MO, USA) at a final concentration of 1 µg/mL was added to the culture medium to induce Tat expression in RPMI-8866 cells. After treatment, cells were kept in the Dox-containing medium for 6, 24 and 48 h. To monitor ROS production, Tat expression was induced in the RPMI-8866 cells by treating with 1 µg/mL doxycycline for 6 h (Dox) in the presence or absence of Tempol (#10-2471, Focus Biomolecules, Plymouth Meeting, PA USA) at a final concentration of 80 µM. Rapamycin (#R-0395, Sigma-Aldrich, Saint Louis, MO, USA) was added to culture medium at a final concentration of 200 nM for 6, 24 and 48 h. Purified and functionally active Tat protein was produced by Advance Bioscience Laboratories (ABL, Rockville, MD, USA) and obtained through the NIH AIDS Research and Reference Reagent Program, USA. According to the manufacturer, the Tat protein was >95% pure after purification by heparin affinity chromatography and reverse-phase HPLC with removal of endotoxins. Tat was added to culture medium at final concentration of 250 ng/mL. Cells were kept in the Tat-containing medium for 6, 24 and 48 h. 

### 4.3. Western Blotting

Cells were collected by centrifugation, washed once with ice-cold 1× PBS and resuspended in the lysis buffer (150 mM NaCl, 1 mM EDTA, 50 mM Tris-HCl, pH 7.5 and 0.5% Nonidet P-40) containing protease inhibitors (#04693159001, Roche, Neuilly sur Seine, France) and phosphatase inhibitors (#04906837001, Roche, France). Cell lysates were kept on ice for 30 min and sonicated for 15 s—5 s on, 3 s off—at 30% amplitude. Protein concentration was quantified using the BCA protein assay kit (#23227, Thermo Fisher Scientific, France) according to the manufacturer’s protocol. Samples were prepared using LDS buffer (#NP0007, Thermo Fisher Scientific, France) and run on 4–12% Bis-Tris gel (#NP0323, Thermo Fisher Scientific, France) in MOPS-SDS buffer (#NP0001, Thermo Fisher Scientific, France) for 30 min at 80 V and for 1 h at 120 V. Separated proteins were transferred onto the PVDF membrane (IPVH00010, Millipore, France) for 2 h at 90 V at 4 °C. Membrane was blocked in 5% milk at room temperature for 1 h. Blocked membrane was incubated with primary antibodies overnight. Nonspecific bindings were washed three times with a washing buffer (0.05 M Tris, 0.15 M NaCl, pH 7.6) containing 0.1% Tween 20. Membrane was then incubated with anti-mouse or anti-rabbit secondary peroxidase-conjugated antibodies for 2 h at room temperature. The membrane was washed three times, incubated with the SuperSignal West Pico chemiluminescent substrate (#34580, Thermo Fisher Scientific, France) for 1 min and then visualized using the ImageQuant LAS 4000 Mini system (GE Healthcare, Vélizy-Villacoublay, France).

Western blot bands on the images obtained from ImageQuant LAS 4000 Mini system were quantified by ImageJ and normalized to the corresponding GAPDH band intensity used as the loading control. Relative band intensities were presented as fold change.

The following antibodies were used: HIV-1 Tat antibody (#sc-65912, Santa Cruz Biotechnology, Heidelberg, Germany); Akt (#9272), phospho-Akt (Ser473) (#9271), p70 S6 Kinase (#9202), phospho-p70 S6 Kinase (Thr389) (#9206), 4E-BP1 (#9452), phospho-4E-BP1 (Thr37/46) (#2855), phospho-Chk1 (Ser345) (#234), phospho-Chk2 (Thr 68) (#26611) and GAPDH (#2118) antibodies were from Cell Signaling Technology, Boston, MA, USA; phospho-Histone H2A.X (Ser139) antibody (#AB_2793161, Active Motif, La Hulpe, Belgium); anti-mouse (#315035003) and anti-rabbit (#111035144) peroxidase-conjugated secondary antibodies were from Jackson ImmunoResearch, Cambridge, UK. 

### 4.4. ROS Production Analysis

To monitor the ROS production, Tat expression was induced either in the presence or in the absence of Tempol. ROS level was detected using the General Oxidative Stress Indicator CM-H2DCFDA (C6827, Thermo Fisher Scientific, France) according to the manufacturer’s guidelines. Briefly, cells were collected, washed once with PBS, resuspended in PBS containing 1 µM of CM-H2DCFDA and incubated at 37 °C for 15 min. Cells were further washed once with PBS and resuspended in PBS. Fluorescence emission spectra of oxidized CM-H2DCFDA at 488 nm were detected by a BD Accuri C6 Plus flow cytometer. 

### 4.5. qRT-PCR

Total RNA was extracted using the NucleoSpin® RNA II kit according to the manufacturer’s recommendations (#740955.250, Macherey-Nagel, Oensingen, Switzerland). Total RNA (300 ng) was primed in a 20 µL cDNA Synthesis Master Mix (#M1661, Thermo Fisher Scientific, France). The obtained cDNA was amplified using specific primers and the PowerUp SYBR Green Master mix (#A25742, Applied Biosystems, Bourgoin-Jallieum, France). Expression of target genes was analyzed with the 2^−ΔΔct^ method by normalizing against *GAPDH* and the expression was compared between different groups. For quantification, expression levels were set to 1 in controls. The primers used for RT-qPCR were designed in Primer3web v. 4.1.0 and are listed in Table S1.

### 4.6. Immunofluorescence

Cells were seeded on Poly-L-lysine-coated 15 × 15 mm coverslips (20 min, 37 °C and 5% CO_2_), rinsed with 0.3× PBS and fixed with 4% paraformaldehyde (Euromedex, Souffelweyersheim, France) in 0.3× PBS for 10 min at room temperature. After washing three times with 1× PBS, coverslips were incubated with 0.5% BSA (Euromedex, France) in 1× PBS for 40 min at room temperature. Then, cells on the coverslips were incubated with the primary antibody against γH2AX (1:500 dilution) for 2 h at room temperature, then washed with PBS 3 times for 5 min and incubated with the secondary antibody conjugated to anti-rabbit Alexa Fluor-488 (Life Technologies, Bourgoin-Jallieum, France 1:200 dilution) for 1 h at room temperature. Nuclei were counterstained with DAPI diluted into the Vectashield mounting medium (Vector Laboratories, Burlingame, CA, USA). Coverslips were mounted on glass slides and further analyzed by a confocal microscope. For visual effect, the Alexa Fluor-488 staining in Figure 1G is represented in red.

### 4.7. Microscope Image Acquisition and Analysis

Immunofluorescence images for γH2AX staining were acquired using a TCS SP8 confocal microscope (Leica Microsystems, Berlin, Germany) with a 63× oil immersion objective. Z-stacks were obtained using a frame size of 1024 × 1024 and 0.5 µm z-steps, with sequential multitrack scanning using 488 nm laser wavelengths. Images were analyzed by ImageJ software.

### 4.8. Statistical Analyses

One-way ANOVA test with Bonferroni’s post-test was used to compare between groups. The Student t-test with two-tailed distribution was used to compare pairs of samples. All tests were conducted on Graphpad Prism v. 5 software (Graphpad software Inc., La Jolla, CA, USA).

## Data Availability

Not Applicable.

## Supplementary Materials

The following are available online at https://www.mdpi.com/1422-0067/22/4/1588/s1.
