# Supplementary material for: HIV-1 Tat Activates Akt/mTORC1 Pathway and AICDA Expression by Downregulating Its Transcriptional Inhibitors in B Cells
Source: Int J Mol Sci. 2021 Feb 4;22(4):1588. doi: 10.3390/ijms22041588 (PMC7915967; doi:10.3390/ijms22041588)
Supplement: Supplementary file 1 [file ijms-22-01588-s001.pdf]

SUPPLEMENTARY MATERIALS

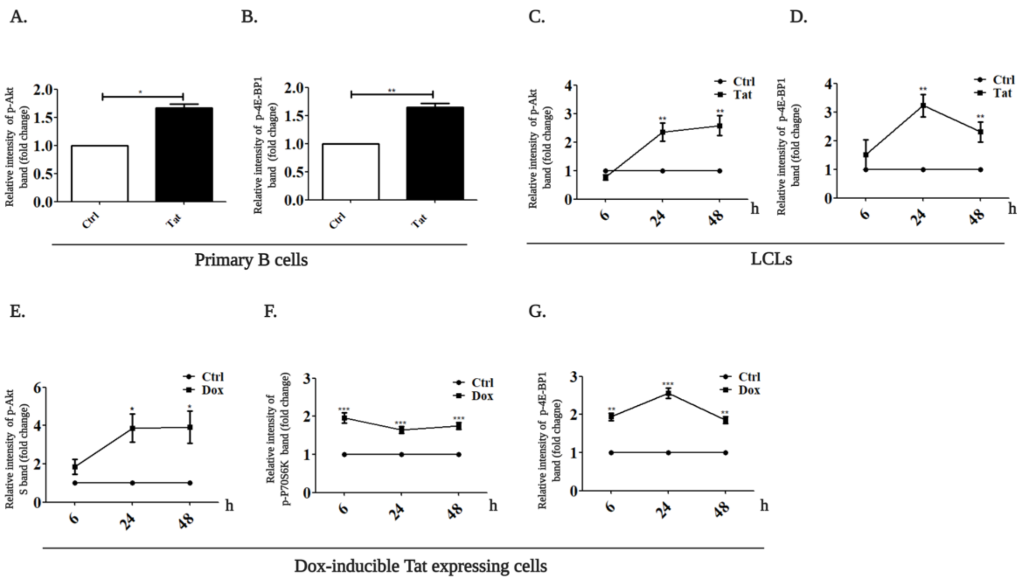

Figure S1. HIV-Tat activates the Akt/mTORC1 pathway in B cells. **A.,B.** Primary B cells purified from the blood of healthy donors were treated (Tat) (or not (Ctrl)) with 250 ng/mL Tat for 48 h. Intensities of p-AKT Ser473 (**A**) and p-4E-BP1 Thr 37/46 (**B**) bands in the Western blots from Figure 2A were quantified with ImageJ software in Tat-treated cells compared to the untreated control (set as 1), after normalization with band intensities of GAPDH (loading control). Relative band intensities on the Y axis are presented as fold change. **C.,D**. Immortalized lymphoblastoid cell lines (LCLs, RPMI-8866) were treated with 250 ng/mL Tat for 6, 24 and 48 h (see Figure 2B). Intensities of p-AKT Ser473 (**C**) and p-4E-BP1 Thr 37/46 (**D**) bands were quantified as described above. **E.,G.** Tat expression in doxycycline-inducible Tat-expressing RPMI-8866 cells was induced (Dox) (or not (Ctrl)) by treating with 1 μg/mL doxycycline for 6, 24 and 48 h (see Figure 2C). Intensities of p-AKT Ser473 (**E**), p-P70S6K Thr389 (**F**) and p-4E-BP1 Thr 37/46 (**G**) bands were quantified as described above. The statistical analyses were carried out by the one-way ANOVA test. All data are expressed as the mean ± SEM. The statistical significance was calculated between groups; ***$p < 0.001$, **$0.001 < p < 0.01$, *$0.01 < p < 0.5$.

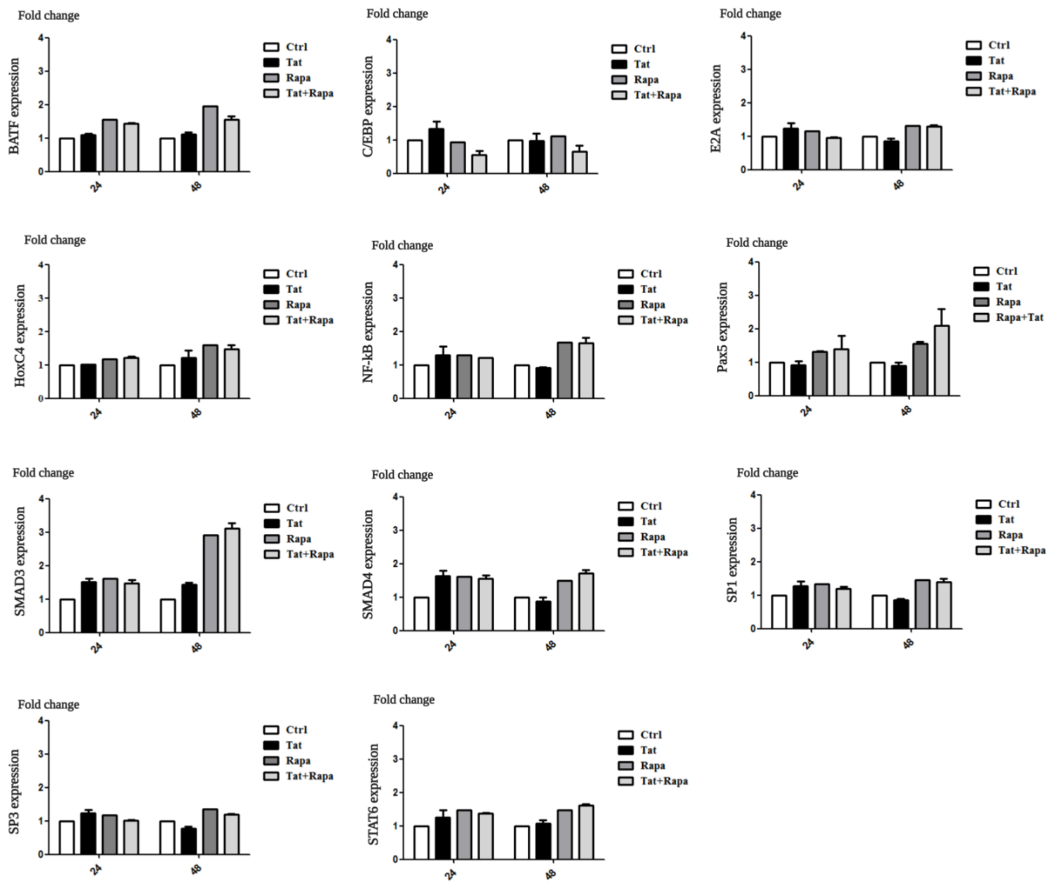

Figure S2. Expression of transcriptional activators of *AICDA* is not affected by Tat induction. mRNA expression levels of *AICDA* activators *BATF*, *C/EBP*, *E2A*, *HoxC4*, *NF-kB*, *Pax5*, *SMAD3*, *SMAD4*, *SP1*, *SP3* and *STAT6* were analyzed by qRT-PCR after 24 and 48 h of Tat expression induction. Expression of target genes in cells treated with 200 nm rapamycin with the untreated control (set as 1) were quantified using the $2^{-\Delta\Delta ct}$ method after normalization with the *GAPDH* gene expression. The data from three independent experiments are represented as the mean ± SEM.

Table S1. List of primers used in this study.

Gene name	Forward primer (5′–3′)	Reverse primer (5′–3′)
PAX5	ACAGCTCTTTCCTTCCCCTC	GGGAAGTTGGGCTAGGTCTT
AICDA	TCTTGATGAACCGGAGGAAG	AGCCGTTCTTATTGCGAAGA
BATF	GCAAGGAGATCAAGCAGCTC	GAGCTGACATGAGGTTGGTG
C/EBPb	TTTTGTCCAAACCAACCGCA	TGCATCAACTTCGAAACCGG
C-Myb	CATTTGATCCGCATCCCCTG	TCAAAAGTTCAGTGCTGGCC
E2A	ATGGGGCATTTTGTTGGGAC	TCCTGTCTACGTCACGATGG
E2F1	GGTCCCTGAGCTGTTCTTCT	CCACTCACCTCTCCCATCTC
E2F2	CTCCTGGGTGAGCTGAAGAA	AAGGAGGCTTACATGGTGCT
E2F3	GGTGGGGTCAAGACAGATGA	ACCAAGTCCAGTGTGTGTGA
E2F4	ACAGTGGTGAGCTCAGTTCA	GAGGTAGAAGGGTTGGGTCC
E2F5	CGGCGTTCTGGATCTCAAAG	TTACAGCCAGCACCTACACC
E2F6	TGTTCCAGCTCCCAGAGAAG	TCTTCTTCCTCAGGGCCTTC
E2F7	CGTCTTTCAGTGTCCCTTGC	TATTGATCCAAGGCCAGGCA
E2F8	GGAGGTGAGACGGTCTTCAA	TGGGAAGGGTGCAGAATTCT
HOXC4	TCCTCTCCCTCCCACTGTTA	AAGCCAGACCATCACACCTT
SMAD3	CTCTGGGTGCTTGGGAACTA	ATCCAAATGCAGCCAAACGT
SMAD4	ACAAGTCAGCCTGCCAGTAT	GGTGCAGTCCTACTTCCAGT
SP1	GAGCAAAACCAGCAGACACA	ACTGTTGGTGTCCGGATGAT
SP3	TGCCTTGGACGTGGATAGC	GCCCTATCTTGCTGCAGGTA
STAT6	AAGAGCACAGGTTAGGGCAT	TAACCACATGTCCAGACCCC
Tat	CTAGACTAGAGCCCTGGAAGCA	TGAGGAGGTCTTCGTCGCT
